# Integrating Mendelian randomization and single-cell RNA sequencing to identify therapeutic targets of baicalin for type 2 diabetes mellitus

**DOI:** 10.3389/fphar.2024.1403943

**Published:** 2024-07-26

**Authors:** Ying-Chao Liang, Ling Li, Jia-Lin Liang, De-Liang Liu, Shu-Fang Chu, Hui-Lin Li

**Affiliations:** ^1^ The fourth Clinical Medical College of Guangzhou University of Chinese Medicine, Shenzhen, China; ^2^ Department of Endocrinology, Shenzhen Traditional Chinese Medicine Hospital, Shenzhen, China

**Keywords:** type 2 diabetes mellitus, baicalin, therapeutic target, Mendelian randomization, single-cell RNA sequencing, traditional Chinese medicine

## Abstract

**Background:**

Alternative and complementary therapies play an imperative role in the clinical management of Type 2 diabetes mellitus (T2DM), and exploring and utilizing natural products from a genetic perspective may yield novel insights into the mechanisms and interventions of the disorder.

**Methods:**

To identify the therapeutic target of baicalin for T2DM, we conducted a Mendelian randomization study. Druggable targets of baicalin were obtained by integrating multiple databases, and target-associated cis-expression quantitative trait loci (cis-eQTL) originated from the eQTLGen consortium. Summary statistics for T2DM were derived from two independent genome-wide association studies available through the DIAGRAM Consortium (74,124 cases vs. 824,006 controls) and the FinnGen R9 repository (9,978 cases vs. 12,348 controls). Network construction and enrichment analysis were applied to the therapeutic targets of baicalin. Colocalization analysis was utilized to assess the potential for the therapeutic targets and T2DM to share causative genetic variations. Molecular docking was performed to validate the potency of baicalin. Single-cell RNA sequencing was employed to seek evidence of therapeutic targets’ involvement in islet function.

**Results:**

Eight baicalin-related targets proved to be significant in the discovery and validation cohorts. Genetic evidence indicated the expression of ANPEP, BECN1, HNF1A, and ST6GAL1 increased the risk of T2DM, and the expression of PGF, RXRA, SREBF1, and USP7 decreased the risk of T2DM. In particular, SREBF1 has significant interaction properties with other therapeutic targets and is supported by strong colocalization. Baicalin had favorable combination activity with eight therapeutic targets. The expression patterns of the therapeutic targets were characterized in cellular clusters of pancreatic tissues that exhibited a pseudo-temporal dependence on islet cell formation and development.

**Conclusion:**

This study identified eight potential targets of baicalin for treating T2DM from a genetic perspective, contributing an innovative analytical framework for the development of natural products. We have offered fresh insights into the connections between therapeutic targets and islet cells. Further, fundamental experiments and clinical research are warranted to delve deeper into the molecular mechanisms of T2DM.

## 1 Introduction

Type 2 diabetes mellitus (T2DM) is a chronic metabolic disorder characterized by inadequate insulin secretion and insulin resistance, leading to dysregulation of glucose homeostasis in the bloodstream. The management and treatment of diabetes pose considerable challenges across all sectors of society. In 2021, the global population of diabetes sufferers of all ages reached 529 million, with over 90% being afflicted by type 2 diabetes (GBD, 2021 Diabetes Collaborators, 2023). Type 2 diabetes imposes a substantial health and economic burden on individuals and the public health system (Sun et al., 2022). Despite the numerous pharmacotherapeutic options available, they frequently possess limited therapeutic efficacy and an abundance of adverse effects (Maruthur et al., 2016). Thus, finding safer and more efficient treatments is crucial for early intervention of T2DM. Baicalin, one of the major active constituents of Scutellaria baicalensis, exhibits favorable pharmacokinetic properties and potent hypoglycaemic effects (Froldi et al., 2022). Several animal studies have suggested that baicalin improves metabolic function in skeletal muscle, adipose tissue, and liver by reducing lipid accumulation and enhancing insulin sensitivity, thus effectively suppressing hyperglycemia and improving insulin action (Yu et al., 2022; Szkudelski and Szkudelska, 2023). Moreover, it saliently reduces hyperglycemia-induced oxidative stress by increasing the activity of antioxidant enzymes and alleviating diabetes-related oxidative damage (Waisundara et al., 2011). Baicalin also inhibits vascular inflammation induced by high glucose levels, suggesting potential therapeutic effects on diabetic vascular complications (Ku and Bae, 2015). In addition, baicalin can alleviate pancreatic fibrosis by blocking the activation of pancreatic stellate cells and ameliorate pancreatic β-cell injury by encouraging beneficial apoptosis (Zhao et al., 2021; Miao et al., 2024). These researches have demonstrated the potential regulatory effects of baicalin on the pancreas, although precise molecular mechanisms and linkages to genetic variation have not been completely elucidated. Insufficient comprehension has limited the further development and application of baicalin as a potential therapeutic agent for diabetes.

The rapid development of genome-wide association studies (GWAS) has identified thousands of genetic variants linked to human diseases and medically essential traits, providing unprecedented opportunities to develop new drugs for complex diseases (Reay and Cairns, 2021). Expression quantitative trait locus (eQTLs) are genetic variations that regulate the expression of specific genes and offer clues for drug discovery (Chauquet et al., 2021). Since most risk variants for complex diseases exert their biological effect by influencing gene expression, integrating GWAS with eQTLs can aid in identifying potential drug targets. Incorporating human genetics and genomics may be one of the most efficient strategies to advance medication research, as therapies supported by genetic evidence are more likely to succeed in clinical trials and gain regulatory approval (Trajanoska et al., 2023). Currently, there is a lack of research investigating complementary and alternative treatments from a genome-wide perspective, and the identification and mechanistic exploration of natural drug targets may offer opportunities for preventing and treating T2DM.

Mendelian randomization (MR) is an epidemiological method that utilizes genetic variants as instrumental variables to assess causal relationships between exposures and outcomes. Its design is based on the random assortment of alleles during gamete formation, which is analogous to a natural randomized controlled experiment. Compared to traditional observational studies, this approach is more effective in minimizing bias due to confounding or reverse causation (Bowden and Holmes, 2019). This sophisticated design can more accurately capture the association between gene expression and complex disease phenotypes, providing a powerful tool for drug target discovery and validation (Võsa et al., 2021). Single-cell transcriptome sequencing technology provides a novel perspective in investigating the pathogenic mechanisms of natural compounds targeting T2DM(Papalexi and Satija, 2018). The application of this technology has already advanced our understanding of disease complexity in several fields and provided important molecular information for personalized medicine (Xin et al., 2016). Compared to bulk RNA sequencing, single-cell RNA sequencing reveals heterogeneity in gene expression within cell populations, enabling more precise identification and targeting of therapeutic targets and enhancing our understanding of specific signaling pathways.

Applying large-scale GWASs and high-throughput single-cell RNA sequencing data, this study systematically integrates various bioinformatics techniques to identify the targets of baicalin in the treatment of type 2 diabetes and elucidate its mechanisms. It innovatively integrates natural products with modern genetics, providing novel insights for drug development and precision medicine.

## 2 Materials and methods

**FIGURE 1:**
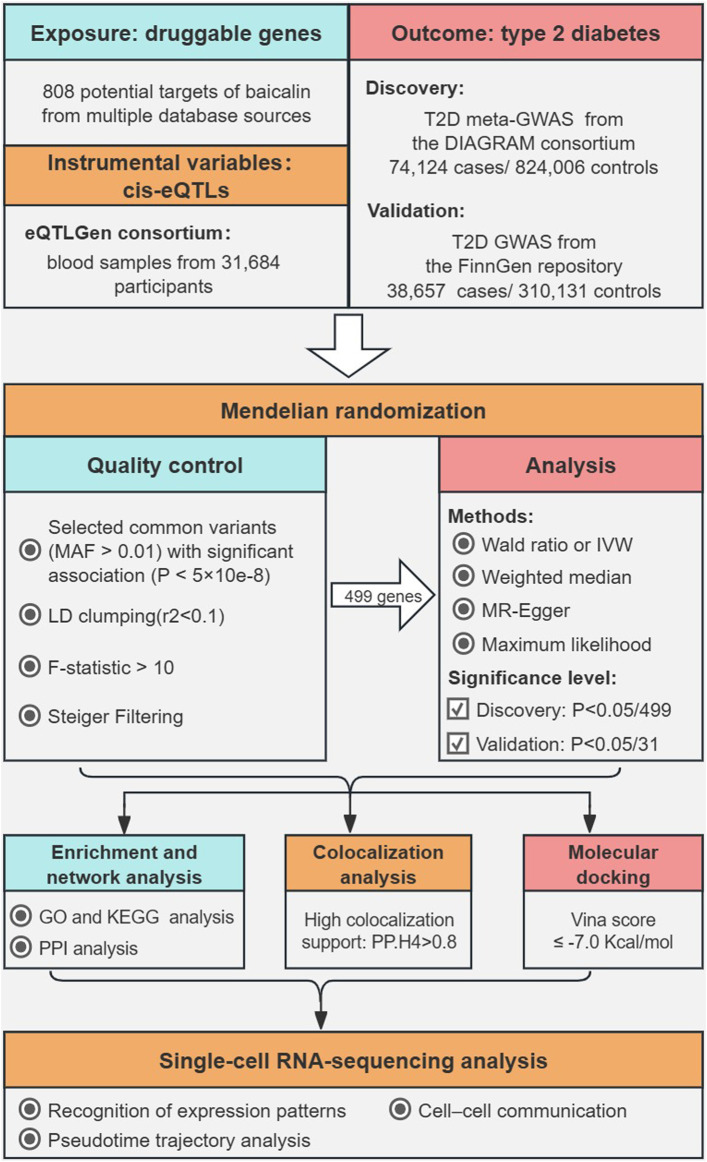
The overall flowchart of this study.

The overall structure of this study based on guidelines for Mendelian randomization research was illustrated in Figure 1 (Burgess et al., 2019; Skrivankova et al., 2021). Potential targets of baicalein were collected *via* multiple sources regarding natural products. We utilized the expression quantitative trait locus (eQTL) related to the drug targets in the eQTLGen Consortium as the exposure and performed MR analysis with two independent T2D cohorts as the outcomes. Then, we conducted functional enrichment analysis and constructed a protein-protein interaction (PPI) network for the therapeutic targets. Bayesian colocalization was employed to assess the possibility of shared causal genetic variation between baicalin targets and T2D, and molecular docking was performed to evaluate the affinity between baicalein and therapeutic targets. Finally, we utilized single-cell RNA sequencing data to explore the potential mechanisms by which therapeutic targets of baicalin are involved in pancreatic function. Subjects included in the discovery and validation cohorts were restricted to European descent to minimize potential bias in population stratification. Data was derived from aggregated meta-GWASs and publicly available eQTL statistics, with original studies authorized by their respective institutional review boards and ethics committees, and all participants granted informed consent.

### 2.1 Data sources for the expression quantitative trait locus

The expression quantitative trait locus (eQTL) refers to genetic variations associated with gene expression levels. It provides accurate proximity to target genes in pharmaceutical research and exerts a more direct regulation on gene expression. The eQTL data in this study originated from the meta-analysis of the eQTLGen Consortium, and a detailed account of the data preparation can be found in the original publication (Võsa et al., 2021). The eQTLGen Consortium conducted an analysis of cis- and trans-expression quantitative trait loci using blood samples from 31,684 healthy European individuals across 37 independent cohorts, and a total of 19,250 genes were involved in the study. Cis-eQTL referred to single-nucleotide polymorphisms (SNPs) within 1 Mb of the gene center and detected in at least two cohorts. The complete original data for cis-eQTL and allele frequency information can be downloaded from the eQTLGen portal (https://eqtlgen.org/).

### 2.2 Acquisition of potential targets for baicalin

In this study, potential drug targets were collected from the BATMAN-TCM 2.0 (http://bionet.ncpsb.org.cn/batman-tcm/) (Kong et al., 2024), SymMap V2 (http://www.symmap.org/) (Wu et al., 2019), TCMIP V2.0 (http://www.tcmip.cn/) (Xu et al., 2019), ChEMBL (https://www.ebi.ac.uk/chembl/) (Barbara et al., 2024), CTD (https://ctdbase.org/) (Davis et al., 2023), and STITCH V5.0 (http://stitch.embl.de/) (Szklarczyk et al., 2016) databases, and some remaining targets were comprehensively supplemented by PharmMapper (https://www.lilab-ecust.cn/pharmmapper/) (Wang et al., 2017), SwissTarget Prediction (http://www.swisstargetprediction.ch/) (Daina et al., 2019), SuperPred (https://prediction.charite.de/) (Nickel et al., 2014) and SEA (https://sea.bkslab.org/) (Keiser et al., 2007). The potential targets of baicalin were aggregated by normalizing the gene symbols and removing non-human, unvalidated, invalid, and duplicate genes through the UniProt database (https://www.uniprot.org/). To generate the genetic instruments for proxy baicalin targets, cis-eQTLs derived from the eQTLGen Consortium were restricted to SNPs within 100 kb upstream and downstream of the drug targets.

### 2.3 Data sources for type 2 diabetes

The genome-wide association studies (GWASs) for type 2 diabetes were from the DIAGRAM consortium and the FinnGen R9 repository. Detailed information regarding subject recruitment and quality control can be found in the original publication (Mahajan et al., 2018; Kurki et al., 2023). In the discovery phase, we selected the largest GWAS meta-analysis of European ancestry, involving 74,124 cases and 824,006 controls, available *via* the DIAGRAM portal (https://diagram-consortium.org/) (Mahajan et al., 2018). The diagnosis of T2D was based on the clinical criteria of the American Diabetes Association or the World Health Organization, supplemented by healthcare registries, usage of antidiabetic medications, and valid self-reporting. Levels of GAD antibodies and fasting C-peptide, early insulin intervention, and family history were used to exclude patients with probable type 1 diabetes. The residual inflation of the summary statistics was corrected through genomic control, and meta-analysis was adjusted for BMI. In the replication analysis, we employed a publicly available summary-level GWAS from the FinnGen R9 repository, including 38,657 cases and 310,131 controls (Kurki et al., 2023). T2D was defined according to the World Health Organization guidelines, with the inclusion and exclusion criteria under the International Classification of Disease (ICD, https://r9.risteys.finngen.fi/) codes, specifically the 9th or 10th revision. The probability of overlap in population selection between the exposure and outcome was minimal.

### 2.4 Mendelian randomization

Mendelian randomization study must fulfill three key assumptions: 1) Instrumental variables (IVs) derived from genetic variation should be tightly associated with the exposure; 2) confounding factors are independent of the selected IVs; 3) The instrumental variables solely impact the outcome through the exposure. We implemented a series of rigorous quality controls for cis-eQTLs to obtain reliable genetic instruments. Firstly, we identified common variants (minor allele frequency >0.01) within a 100 kb region surrounding the potential targets of baicalin with a significant threshold of *p* < 5e−8, ensuring that the instrumental variables could serve as proxies for exposure (Chen et al., 2022; Li et al., 2023). Secondly, utilizing a reference panel from the European population of the 1000 Genomes Project (1000 Genomes Project Consortium et al., 2015), we applied a linkage disequilibrium-based clustering with an *r* ^2^ = 0.1 threshold within a 10,000 kb range to eliminate potential confounding effects generated by linkage between SNPs(Chen et al., 2022). Thirdly, we computed the F-statistic for each instrumental variable to estimate their strength (*R* ^2^ = 2×EAF×(1-EAF) × beta^2^; F = *R* ^2^ × (N-2)/(1-R^2^)) and excluded SNPs with an F-statistic below 10 to eliminate bias from weak instrumental variables (Burgess et al., 2011; Papadimitriou et al., 2020). Fourthly, Steiger filtering was applied to remove drug targets where SNPs accounted for a larger fraction of the variation in T2D risk than gene expression to ensure unidirectionality of causality. In addition, we removed palindromic SNPs with uncertain strands and SNPs with non-concordant alleles to avoid any potential errors in allele determination and provide accurate causality assessments.

We utilized the “TwoSampleMR” package (version 0.5.7) in R software (version 4.2.1) for the MR procedure and sensitivity analysis (Hemani et al., 2018). In the primary analysis, if only one eQTL for the drug target was available, we employed the Wald method that calculated the coefficient ratio for the outcomes and exposures. When two or more instrumental variables were available, the meta-analysis integrated with the Wald ratio on each SNP was performed using inverse-variance weighted (IVW), MR-Egger, weighted median, and maximum likelihood methods. The primary assessment was completed by applying the IVW approach, yielding general estimates through meta-analysis in combination with Wald ratios for each SNP (Burgess et al., 2017). Compared to the fixed effects model, The IVW method with multiplicative random effects model (REM) could guarantee statistical efficacy even in the presence of weaker random effects (Burgess et al., 2019). In comparison to the IVW approach, the other methods exhibited relatively inferior statistical efficacy. Consequently, they were solely applied to corroborate the general direction of the primary method.

We applied the IVW method and Egger regression to detect heterogeneity for baicalin targets containing two or more instrumental variables. Heterogeneity was quantified using Cochran’s Q test, with *p* < 0.05 indicating apparent heterogeneity among instrumental variables. The MR-Egger intercept was employed to evaluate the existence of pleiotropy across instrumental variables, and no meaningful horizontal pleiotropy was observed if *p* > 0.05. Bonferroni correction was implemented in the discovery cohort to define the significance threshold for multiple testing. Drug targets with a *p*-value <1.00e-4 (0.05/499) were defined as significant. Sensitivity analysis was conducted on the initially identified potential targets, and validation was performed in a replication cohort. The significance threshold for the validation phase was established at 0.0016 (0.05/31) (Cao et al., 2023).

### 2.5 Network construction and functional enrichment analysis

Enrichment analysis of gene clusters was performed *via* the R package “clusterProfiler” (version 4.4.4) to identify biological pathways for potential targets of baicalin. Gene Ontology (GO) enrichment analysis elucidates the biological significance of genes from three perspectives: biological processes (BP), cellular components (CC), and molecular functions (MF). The R package “org.Hs.e.g.,.db” is used for gene ID conversion. Kyoto Encyclopedia of Genes and Genomes (KEGG) pathway enrichment analysis was performed through the KOBAS-i portal (http://bioinfo.org/kobas/) (Bu et al., 2021). To explore potential interactions between therapeutic targets, the STRING database (version 12.0, https://string-db.org/) was employed to construct Protein-Protein Interaction (PPI) networks.

### 2.6 Bayesian colocalization analysis

To further ascertain the potential shared genetic effects between drug targets and T2D risk, we conduct colocalization analysis using the R package “coloc” (version 5.2.3). Colocalization analysis requires the inclusion of all SNPs within a genomic region, providing a comprehensive method for utilizing genetic information to evaluate the therapeutic targets of baicalin (Wallace, 2020). We applied a prior probability of 1e-04 for baicalein targets (H1) and T2D phenotypes (H2) while setting the prior probability to 1e-05 for an individual variant being associated with both gene expression and T2D risk (H4). For each potential target of baicalin, we included SNPs within a range of ±1 Mb from the gene start and endpoints. The significance criterion for colocalization was defined as PP.H4 greater than 0.80.

### 2.7 Molecular docking

Molecular docking serves to evaluate the binding characteristics of active compounds and therapeutic targets within their three-dimensional structures, and it is widely utilized in drug discovery (Pinzi and Rastelli, 2019). We retrieved the 3D crystal structure of the drug target in PDB format from the RCSB Protein Database (https://www.rcsb.org/), while the 3D chemical structure of baicalin in SDF format was acquired from the PubChem database (https://pubchem.ncbi.nlm.nih.gov/). The CB-DOCK2 web server (https://cadd.labshare.cn/cb-dock2/php/index.php) was applied to validate the binding strength of baicalin with the therapeutic targets (Yang X. et al., 2022; Liu et al., 2022). The core of CB-Dock2 adopts the open-source software, AutoDock Vina. Since the binding sites for ligands are typically large cavities, the docking center, and size are pre-determined based on the identified cavity pockets (C1-C5). After completing the docking process, the binding poses are re-sorted based on Vina scoring. The Vina score indicates the degree of binding between the drug and the protein, with lower scores indicating better binding. The optimal binding site for the active ingredient and the first conformational pose based on the optimal affinity are considered the best binding forms.

### 2.8 Quality control, cluster analysis and identification of cell types for single cell expression data

The dataset GSE153855 was originated from the Gene Expression Omnibus (GEO) data repository and generated at the Science for Life Laboratory in Stockholm using single-cell genomics facilities, and it encompassed pancreatic islet tissues from 5 T2D donors and six control individuals (Ngara and Wierup, 2022). We utilized the R package “Seurat” (version 5.0.1) to process single-cell transcriptomic sequencing data (Hao et al., 2023). Quality control was performed by assessing gene counts, expressions, and the percentage of mitochondrial genes in the sequencing data. We applied nFeature_RNA > 2,000, nCount_RNA > 10,000, and percent.mt < 5 as the threshold for cell selection, and violin plots to demonstrate gene counts, gene expressions, and the percentage of mitochondrial genes. The data was subjected to normalization, feature selection, and standardization. Then it was reduced in dimensionality using principal component analysis (PCA) and clustered and visualized using the t-distributed stochastic neighbor embedding (t-SNE) algorithm. The cell types in the clustering were annotated using the intrinsic information from the dataset.

### 2.9 Inference of intercellular communication

The R package “CellChat” (version 1.6.1) is an appliance that quantitatively infers and analyzes cell-cell communication networks from single-cell expression profiles, and its database (http://www.cellchat.org/) contains known ligand-receptor and their cofactor interactions (Jin et al., 2021). We identified significant ligand-receptor pairs by employing differentially overexpressed genes (*p* < 0.05) across cell clusters, and multiple communication patterns and pathways among cell clusters were categorized by network analysis tools and pattern recognition methods. To elucidate how cell clusters and signaling pathways coordinate and drive intercellular communication, we utilized the “identifyCommunicationPatterns” function to infer the functional specificity of communication patterns.

### 2.10 Pseudotime trajectory analysis

The R package “Monocle” (version 2.22.0) was employed to arrange cells along a hypothetical developmental timeline to infer the differentiation process of cell clusters (Qiu et al., 2017). Utilize the “dispersionTable” function to identify genes with high variability, and employ the “setOrderingFilter” function to pseudo-temporally order the cells. Genes with high variability were identified *via* the “dispersionTable” function. Cells were pseudo-temporally sorted applying the “setOrderingFilter” function, with a threshold of mean_expression≥0.1 and dispersion_empirical≥1 * dispersion_fit for gene inclusion in the ordering. The “DDRTree” method was utilized for dimensionality reduction, and the “orderCells” function was applied to estimate the cell arrangement along the trajectory.

## 3 Results

### 3.1 Acquisition of potential targets for baicalin

We acquired potential targets of baicalin from multiple sources to ensure the comprehensive scope of the study. In particular, there were 58 baicalin-related targets in the BATMAN-TCM 2.0 database, 86 targets in the SymMap V2, 66 targets in the TCMIP v2.0, 258 targets in the ChEMBL, 53 targets in the CTD, 28 targets in the STITCH, 297 targets in the PharmMapper, 100 targets in the SwissTarget Prediction, 94 targets in the SuperPred, and 36 targets in the SEA. After merging, deduplication, and standardization, there are a total of 808 potential drug targets for baicalin. We obtained cis-eQTLs tightly associated with baicalin targets in a reliable (P < 5e×10^−8^) and independent (*r* ^2^ < 0.1, kb = 10,000) manner from the eQTLGen consortium. After a series of quality control measures including exclusion based on F-statistics and Steiger’s filtering, we selected the eQTL from the final set of 499 target genes.

### 3.2 Association of baicalin-related targets with T2D in the discovery cohort

**FIGURE 2:**
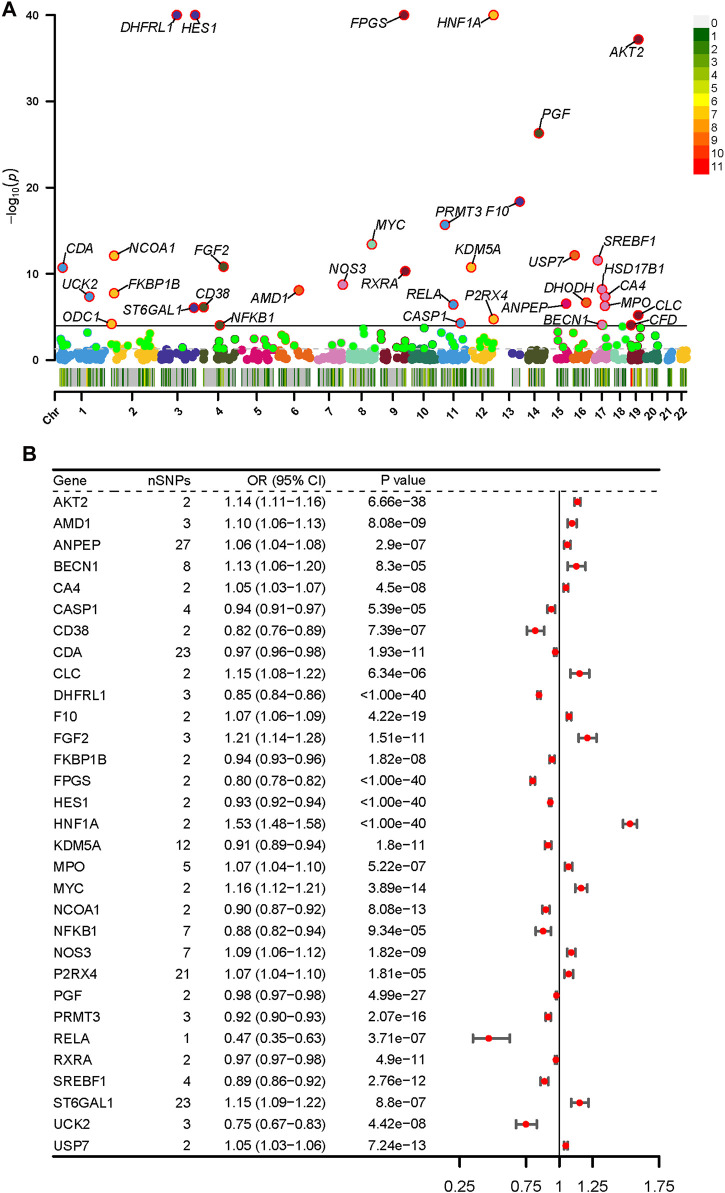
Potential targets of baicalein significantly associated with T2D in the discovery cohort. **(A)** The Manhattan plot of MR analysis at the discovery phase. The dashed line represented a nominal *p*-value (0.05), and the solid line referred to a *p*-value (1.00e−4) adjusted for Bonferroni correction. Significant genes were annotated with labels. **(B)** 31 baicalein-related targets approved by sensitivity analysis. The effect estimates were represented by odds ratios (ORs) and their corresponding confidence intervals (CIs).

During the discovery phase, we selected the largest T2D GWAS currently available as the outcome and conducted MR analysis using eQTLs for potential targets of baicalin. Applying the Wald ratio or IVW method with a multiplicative random effects model, a total of 35 baicalin targets remained significantly (*p* < 1.00e-4) associated with T2D risk after Bonferroni correction (Figure 2A). DHODH, HSD17B1, and ODC1 were excluded from the MR-Egger method due to inconsistent causal estimates in the MR-Egger compared with other methods (Supplementary Table S1). CFD was excluded due to unaccountable heterogeneity. Despite the presence of heterogeneity in ANPEP, BECN1, P2RX4, and ST6GAL1, their causal estimates remained significant after multiple tests in the weighted mean method (*p* < 1.00e-4), indicating relatively robust results (Supplementary Table S2). The Egger intercept test demonstrated no apparent horizontal pleiotropy. After the above sensitivity analysis, 31 genes were included for subsequent validation (Figure 2B). Genetic prediction indicated elevated levels of AKT2, AMD1, ANPEP, BECN1, CA4, CLC, F10, FGF2, HNF1A, MPO, MYC, NOS3, P2RX4, ST6GAL1 and USP7 were associated with increased risk of T2D, while the concentrations of CASP1, CD38, CDA, DHFRL1, FKBP1B, FPGS, HES1, KDM5A, NCOA1, NFKB1, PGF, PRMT3, RELA, RXRA, SREBF1, and UCK2 exhibited a negative correlation with T2D risk. These associations are consistent across other approaches, and the results of genome-wide MR in the discovery phase are presented in Supplementary Table S1.

### 3.3 Association of baicalin-related targets with T2D in the validation cohort

**FIGURE 3:**
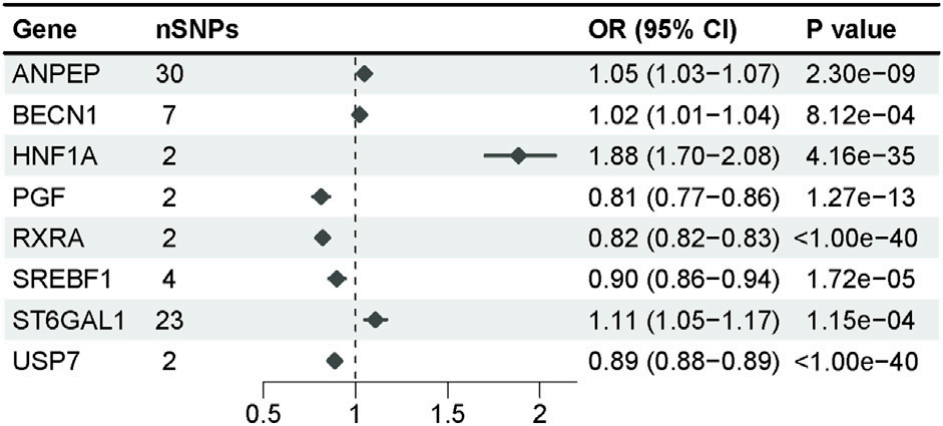
The forest plot showing statistically significant targets after multiple tests (*p* = 0.0016) in the validation cohort.

Employing GWAS from the FinnGen R9 repository for validation, we conducted a replication analysis of the potential targets of baicalin and performed MR analysis in a manner consistent with the discovery cohort. We evaluated the potential causal relationship between potential targets of baicalein and the risk of T2DM by applying the Wald ratio or the IVW method with a multiplicative random effects model (Supplementary Table S3), and 8 baicalin-related targets remained significant (*p* < 0.0016) after Bonferroni correction (Figure 3). Specifically, the elevated expression of ANPEP, BECN1, HNF1A, and ST6GAL1 was associated with an increased risk of T2D, while the expression of PGF, RXRA, SREBF1, and USP7 decreased the risk of T2D. These targets exhibited identical causal effects across the 4 MR methods, and their impact on the outcome in the validation cohort remained consistent with that observed in the discovery cohort. ST6GAL1 demonstrated heterogeneity among SNPs in Cochran’s Q test, yet it exhibited statistical significance in the weighted mean method (Supplementary Table S4). The Egger intercept test revealed no significant pleiotropy for any target.

### 3.4 Functional enrichment and protein-protein interaction analysis of baicalin targets

**FIGURE 4:**
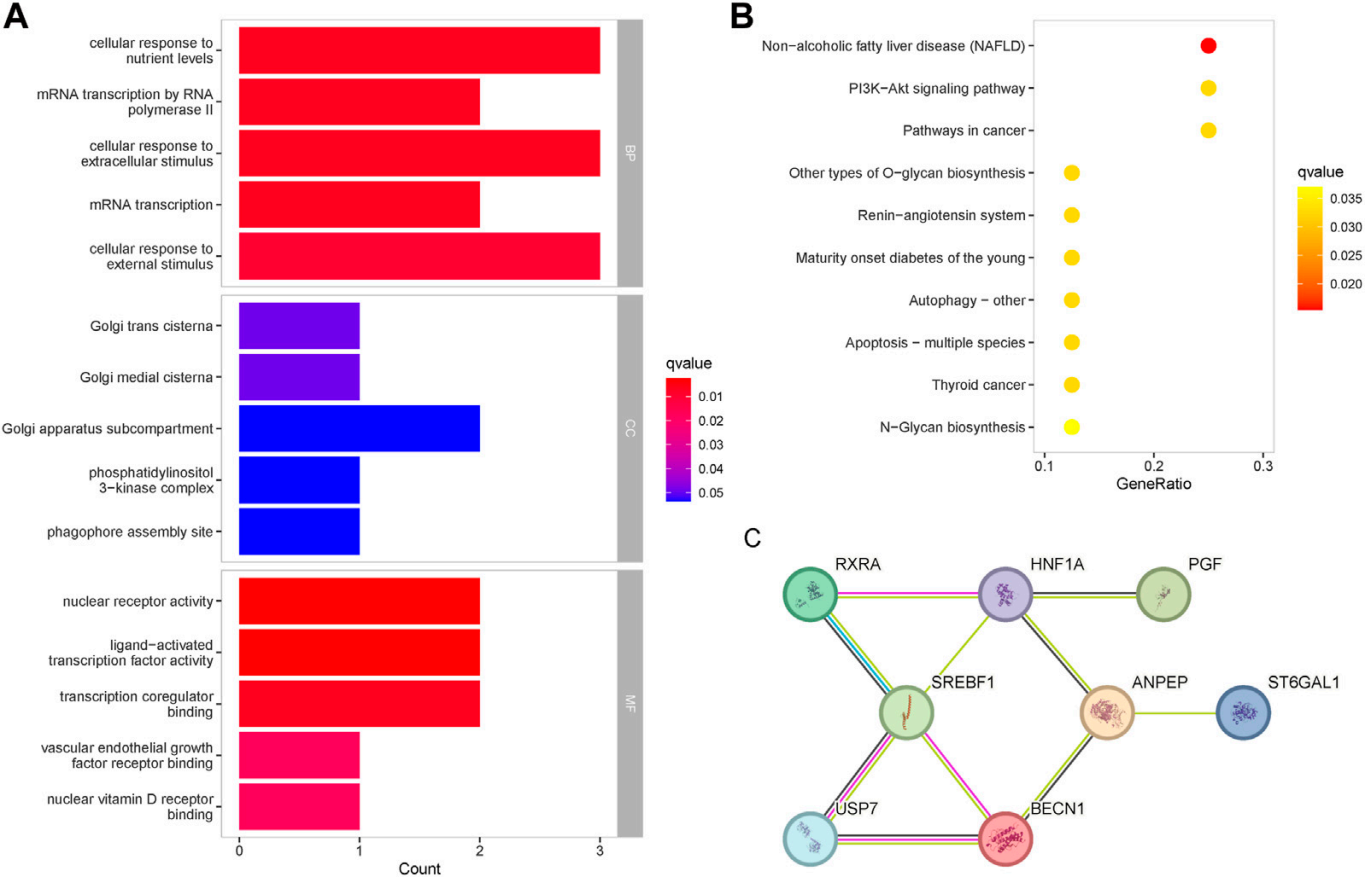
Enrichment analysis of the therapeutic targets for baicalin. **(A)** GO functional enrichment analysis of the targets, ranked according to the adjusted *p*-value, with the top five entries visualized. The horizontal axis represented the number of genes in enrichment, and the color of the bar represented the adjusted *p*-value. **(B)** KEGG pathway enrichment analysis of the therapeutic targets. **(C)** Protein-protein interaction network of the therapeutic targets.

GO enrichment analysis was performed to determine the biological functions of baicalein targets (Figure 4A). The most important enrichment entries for biological processes were: cellular response to nutrient levels, mRNA transcription by RNA polymerase II, cellular response to extracellular stimulus, mRNA transcription, and cellular response to external stimulus, mainly involving mRNA transcription and signal transduction. The most important enrichment entries for molecular function were: nuclear receptor activity, ligand-activated transcription factor activity, transcription coregulator binding, vascular endothelial growth factor receptor binding, and nuclear vitamin D receptor binding. Cellular components were mainly enriched in the Golgi apparatus. The enrichment entries related to diabetes in the KEGG pathway included: the PI3K-Akt signaling pathway, mature onset diabetes of the young, autophagy, apoptosis, and N-Glycan biosynthesis (Figure 4B). PPI analysis revealed that the 8 therapeutic targets shared a tight association, and the targets with the highest centrality degree in the network were SREBF1 and HNF1A (Figure 4C). There were reliable interactions between SREBF1 and RXRA, SREBF1 and USP7, as well as USP7 and BECN1, and suggesting co-expression existed among them.

### 3.5 Colocalization analysis of baicalin-related targets

**FIGURE 5:**
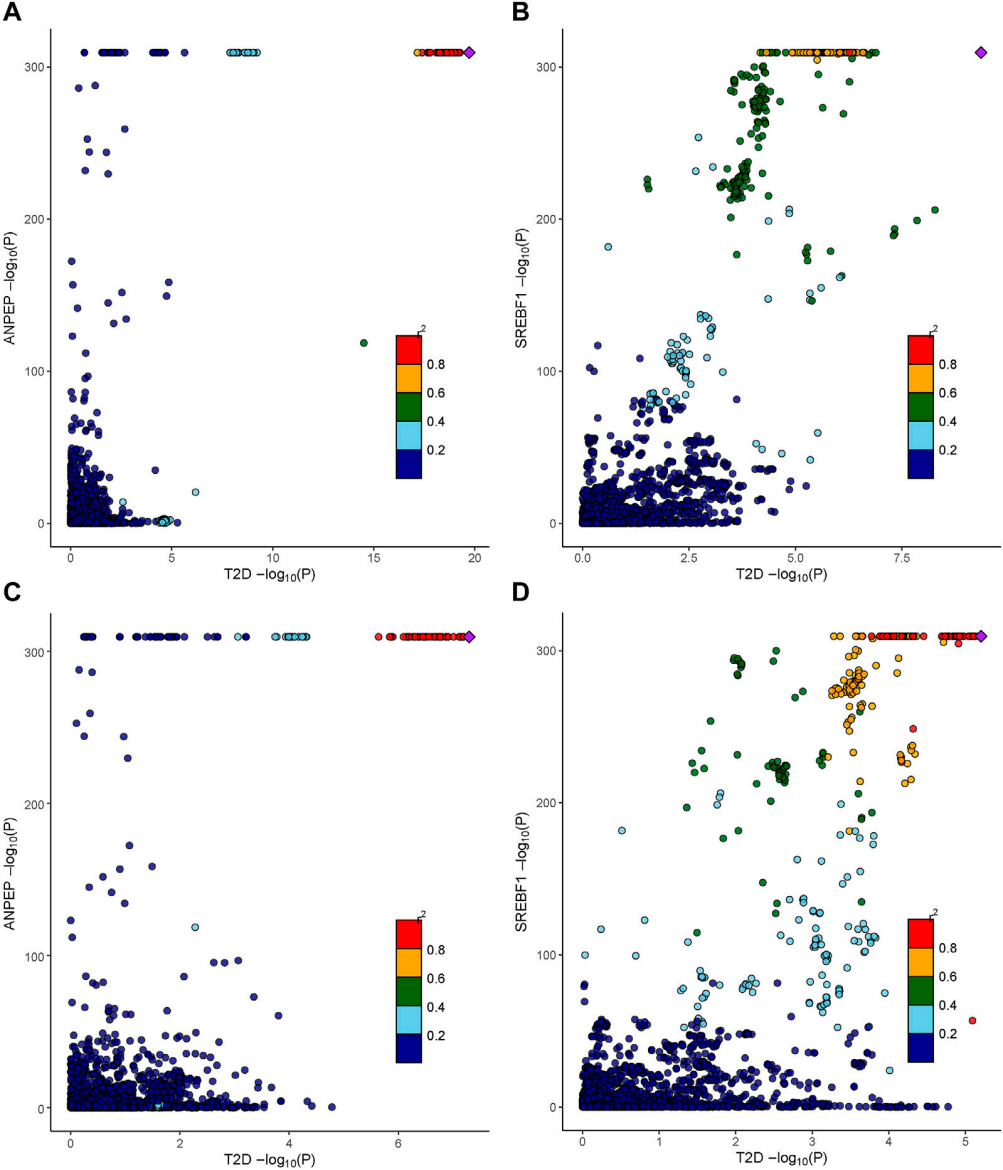
Colocalization analysis results of therapeutic targets for baicalin. **(A)** The association between cis-eQTLs of ANPEP and SNPs of T2D in the DIAGRAM consortium. **(B)** The association between cis-eQTLs of SREBF1 and SNPs of T2D in the DIAGRAM consortium. **(C)** The association between cis-eQTLs of ANPEP and SNPs of T2D in the FinnGen R9 repository. **(D)** The association between cis-eQTLs of SREBF1 and SNPs of T2D in the FinnGen R9 repository.

We further determined the potential causal genetic variants shared between the T2D risk and baicalin targets through colocalization analysis (Figures 5A–D). In the meta-GWAS from the DIAGRAM, ANPEP (PP.H4 = 0.88) and SREBF1 (PP.H4 = 0.85) might share a causal variant with the T2D trait at a genetic locus. In the GWAS from the FinnGen R9, ANPEP (PP.H4 = 0.89), SREBF1 (PP.H4 = 0.83), and PGF (PP.H4 = 0.83) might share a causal variant within the genetic locus with the T2D trait (Supplementary Table S5).

### 3.6 Molecular docking of baicalin-related targets

We retrieved the structural files of 8 therapeutic targets from the RCSB Protein Database and further explored the binding properties of the therapeutic targets with baicalin. We performed molecular docking of the receptor and ligand *via* CB-Dock2, and the Vina scores were demonstrated in Table 1. All paired binding energies were not greater than −7.0 kcal/mol, indicating that baicalin could combine effectively with the therapeutic targets and exert its effects. In particular, the binding energy of RXRA with baicalin was the lowest (−10.7 kcal/mol). The binding energy of ANPEP with baicalin was −9.8 kcal/mol, while USP7 was −9.2 kcal/mol, ST6GAL1 was −9.0 kcal/mol, BECN1 was −8.7 kcal/mol, HNF1A was −8.7 kcal/mol, PGF was −8.1 kcal/mol, and SREBF1 was −7.0 kcal/mol.

**TABLE 1:** Molecular docking results of baicalin with the therapeutic targets.

Ligand	Target	PDB ID	Vina score	CurPocket ID	Cavity volume (Å3)	Center (x, y, z)	Contact residues
Baicalin	ANPEP	4FYQ	−9.8	C4	846	108, 4, 10	Chain A: ASP188 ARG195 SER196 GLU197 TYR198 ARG204 ASP348 PHE816 ARG817 VAL822 ALA825 ASP826 ARG829 PRO851 ASP852 LEU853 ARG855 GLN857 ASP858
Baicalin	BECN1	4DDP	−8.7	C3	202	16, 4, 23	Chain A: ARG292 TRP300 GLY356 LEU357 ARG358 PHE359 LYS416 THR417 GLN418 PHE419 ASN420 SER421 GLU422 TRP425
Baicalin	HNF1A	1IC8	−8.7	C3	464	51, 10, 30	Chain A: GLU90 ASN91 LEU92 SER93 GLU96 ALA97 HIS99 GLN100 VAL103 VAL167 ARG168 LYS169 GLN170 ARG171 VAL173 ALA174
Baicalin	PGF	1FZV	−8.1	C1	175	8, 48, −15	Chain A: CYS66 THR67 GLY68 CYS69 CYS70 GLY71 ASP72 GLU73 LEU75 HIS76 CYS77; Chain B: LEU41 VAL42 ASP43 HIS54 PHE56 SER59 CYS60
Baicalin	RXRA	5K13	−10.7	C1	1941	56, −8, 12	Chain A: TRP225 PHE228 SER229 GLU230 LEU231 SER232 THR233 CYS235 ILE236 LEU266 LEU269 ILE270 ARG272 ILE273 ARG276 PHE286 SER287 PHE302 VAL395 PRO407 PRO408 LEU409 ILE410 MET413
Baicalin	SREBF1	1AM9	−7	C1	166	40, 47, 178	Chain C: GLU332 TYR335 ARG336 ILE339; Chain D: HIS328 GLU332 TYR335 ARG336 SER337 ILE339 ASN340 ILE343 LYS359
Baicalin	ST6GAL1	6QVS	−9	C3	633	25, 8, 74	Chain A: ASN212 GLY213 ASN233 GLN235 LEU236 THR239 GLU240 ARG242 ASP246 LEU248 TYR249 TYR354 TYR356 GLN357 LYS358 PHE359 ASP361 ALA363 CYS364
Baicalin	USP7	5N9T	−9.2	C4	641	−9, 12, 49	Chain B: TYR224 ASP295 VAL296 GLN297 GLN351 GLN405 LEU406 MET407 ARG408 PHE409 MET410 TYR411 ASN418 LYS420 HIS456 ASP459 ASN460 HIS461 TYR465 TYR514

### 3.7 Expression pattern of the target genes in pancreas cells

**FIGURE 6:**
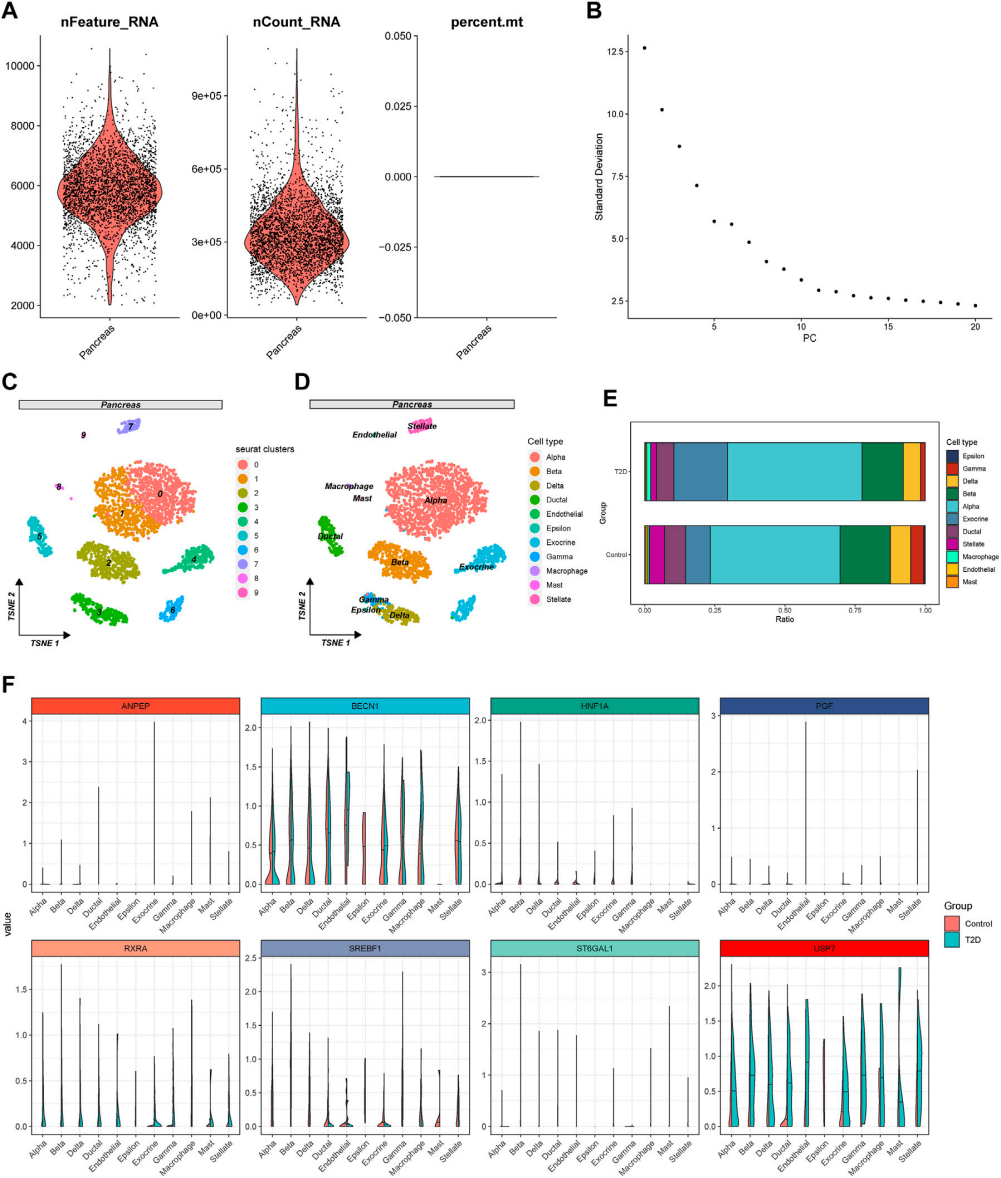
Quality control, clustering analysis, and cell annotation of pancreatic samples. **(A)** Violin plots of gene counts (nFeature_RNA), gene expressions (nCount_RNA), and the percentage of mitochondrial genes (percent. mt). **(B)** The elbow plot of PCA clustering. **(C)** Cell clustering plot of pancreatic tissue under t-SNE dimensionality reduction. **(D)** Annotation plot of cell types for each cluster under the t-SNE algorithm. **(E)** Bar plot of cell proportions in the T2D and control group. **(F)** Violin plots of the differential expression of baicalin-related targets in the two groups of all cell types.

Quality control for the single-cell sequencing dataset GSE153855 was conducted by gene counts and RNA expression as well as the percentage of mitochondrial genes, and we filtered out 19 cells due to inadequate quality (Figure 6A), and excluded 28 cells of unknown type. Utilizing the top 10 principal components (Figure 6B), we performed cell clustering through t-SNE dimensionality reduction to visualize the overall distribution of the data (Figure 6C). Cell types were annotated for each cluster using the integrated annotations in the GSE153855 dataset (Figure 6D). The final 3,336 cells passed the quality control, comprising 1,638 cells from T2D patients and 1,698 cells from the control group. The proportion of most cell types in T2D patients decreased compared to that in the control group, except alpha cells (Control: 46.2%, T2D: 47.9%), exocrine cells (Control: 8.8%, T2D: 19.1%), and macrophage cells (Control: 0.5%, T2D: 1.5%) had increased (Figure 6E). Beta cells accounted for 14.7% of T2D patients, while they accounted for 18.0% in the control group. Endothelial cells (Control: 0.7%; T2D: 0.4%), epsilon cells (Control: 0.5%; T2D: 0.0%), gamma cells (Control: 4.7%; T2D: 1.7%), stellate cells (Control: 5.4%; T2D: 2.0%), and mast cells (Control: 0.4%; T2D: 0.2%) exhibited a salient decrease. The change in cell ratios indicated altered pancreatic function and microenvironment in T2D patients. We found varying degrees of differences in the expression of 8 targets between individuals with T2DM and the control group (Figure 6F). The expression of USP7, RXRA, and BECN1 was highly elevated in pancreas cells, with USP7 and RXRA significantly upregulated in most cell clusters of T2D samples. SREBF1 and HNF1A exhibited higher expression levels in the majority of cell clusters in the control group, while ANPEP, PGF, and ST6GAL1 were predominantly expressed only in specific cell clusters.

**FIGURE 7:**
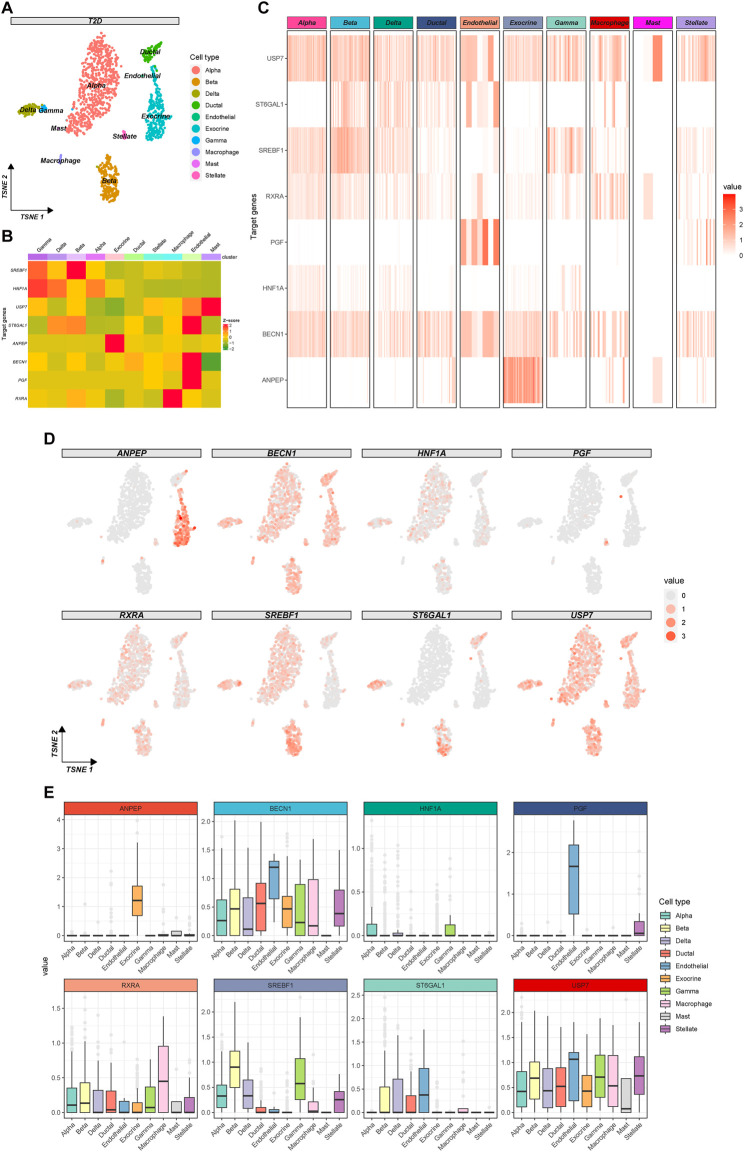
Expression profile of the therapeutic targets of baicalin in T2D samples. **(A)** The clustering plot of the t-SNE algorithm colored by cell types. **(B)** Heatmap for the average expression of target genes in cell clusters. **(C)** Heatmap for the gene expression in each cell. **(D)** The clustering plots of t-SNE algorithm showing gene expressions in each cell. **(E)** Box plots of cell types showing the differential expression of the target genes.

To further explore the potential mechanism of baicalin involvement in the progression of T2D, we utilized t-SNE clustering exclusively for T2D samples (Figure 7A). The heatmap demonstrated the expression of baicalin-related targets in different cell types (Figure 7B) and in each cell (Figure 7C), and the eight targets have a wide range of expression in the pancreatic cells. In particular, SREBF1and ST6GAL1 were highly expressed in beta cells, so were HNF1A and SREBF1 in gamma cells, ANPEP in exocrine cells, RXRA in macrophage cells, ST6GAL1, BECN1 and PGF in endothelial cells, and USP7 in mast cells. Figure 7D provided a detailed display about the expression of the targets in each cell under the t-SNE algorithm, and Figure 7E illustrated the differential expression of the targets in pancreatic cell clusters.

### 3.8 Cell-cell communication

**FIGURE 8:**
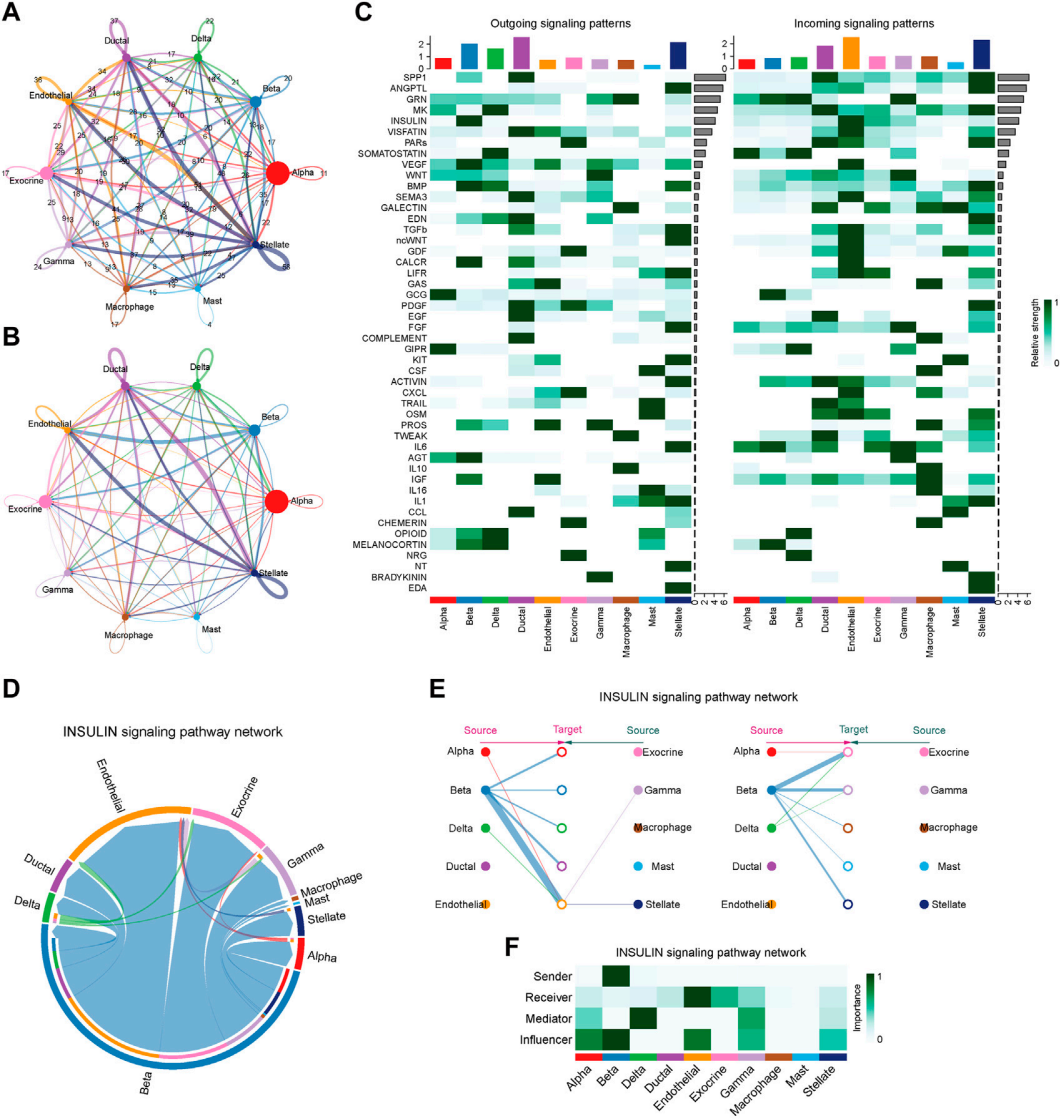
Intercellular communication and signaling pathway analysis on T2D samples. **(A)** The plot of intercellular communication is presented by the number of interactions. The thickness of the line was proportional to the number of ligands. **(B)** Plot of intercellular communication presented by the weights of interactions. The thickness of the line was proportional to the weight of the interactions. **(C)** Heatmap depicting the contribution of outgoing and incoming pathways to intercellular communication. **(D)** Circular diagram illustrating the interactions of the insulin signaling pathway across cell clusters. **(E)** Relationship diagram depicting the interactions of the insulin signaling pathway in cell clusters. **(F)** Heatmap showing the involvement of pancreatic cells in the transduction of the insulin signaling pathway.

To characterize the islet microenvironment in T2D patients, CellChat was employed to infer and quantify the interactions between cell clusters. The findings indicated that ligand-receptor pairs exhibited extensive molecular interactions among 10 pancreatic cells, and stellate, endothelial, ductal, and beta cells shared strong interactions (Figures 8A, B). The top five contributing outgoing and incoming pathways for intercellular communication were the SPP1, ANGPTL, GRN, MK, and insulin signaling pathways. We further explored the insulin signaling pathway involved in the function of the pancreatic cells (Figure 8D). The intercellular communication between beta cells and endothelial or exocrine cells had a significant impact on the transduction process of the insulin signaling pathway (Figure 8E). Beta cells were responsible for the transmission of the insulin signal pathway, while endothelial and exocrine cells were primarily involved in signal reception. Delta cells could play a certain intermediary role, and alpha, beta, and endothelial cells could influence signal transduction (Figure 8F).

### 3.9 Pseudotime trajectory analysis

**FIGURE 9:**
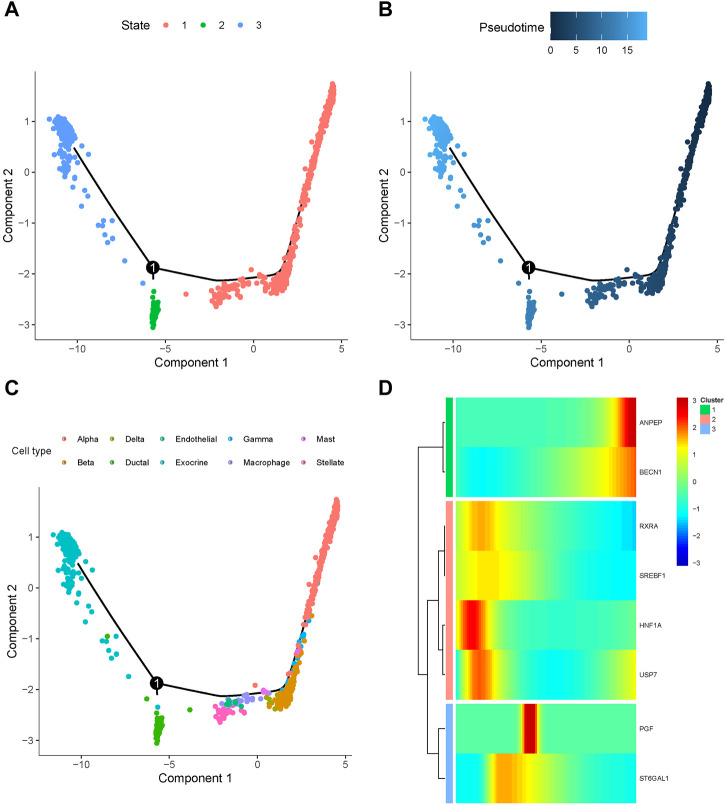
Differentiation trajectory of simulated cell development in T2D samples. Dot plots of cell trajectories are colored based on cell state **(A)**, pseudotemporal order **(B)**, and cell type **(C)**, with each dot corresponding to a single cell. **(D)** Heatmap for the expression of baicalein therapeutic target in single cells arranged by pseudotemporal order, with colors from blue to red indicating relative expression levels from low to high.

To further elucidate the mechanisms underlying the involvement of baicalein targets in pancreatic cell development of T2D progression, we employed Monocle to categorize these genes at the single-cell transcriptomes and constructed a dendritic structure of the entire lineage differentiation trajectory. Cell trajectory was colored according to the states of cell populations, pseudotime progression, and cell types (Figures 9A–C). As the motor trajectory progresses, the pancreatic cells transition through three states: the branch initiation point (pre-branching) and two other branches. Alpha primarily appeared in the initial stage of the trajectory before branching, and gamma, beta, delta, mast, macrophage, endothelial, and stellate cells completed the differentiation over a short period of time. Ductal and exocrine cells mainly appeared at the two ends of the trajectory after branching. The heatmap displayed the expression trends of the baicalin-related genes along the pseudotime trajectory, classifying them into three clusters with distinct expression dynamics (Figure 9D). The expression of RXRA, SREBF1, HNF1A, and USP7 initially surged and then gradually diminished along the pseudotime trajectory, while ANPEP and BECN1 peaked at their maximum expression levels at the completion of differentiation, and the expression of PGF and ST6GAL1 exhibited a tidal wave trend along the pseudotemporal trajectory. We delineated the orderly and progressive trajectory of pancreatic cell development in T2D samples and depicted the dynamic gene expression of baicalin therapeutic targets along the pseudotemporal trajectory. This might reveal the pseudo time dependence of these genes in the formation and development of pancreatic cells, as they acted upon specific cellular differentiation cycles and exerted an impact on disease progression.

## 4 Discussion

Applying eQTLs for baicalin-related targets, we performed MR analysis in discovery and replication cohorts and identified eight therapeutic targets causally associated with T2DM: ANPEP, BECN1, HNF1A, ST6GAL1, PGF, RXRA, SREBF1, USP7. SREBF1, known as SREBP1, engages in the encoding of sterol regulatory element binding proteins. With strong colocalization support, SREBF1 was highly expressed in β-cells and exhibited important interacting properties in the PPI network. SREBP1c, one of the transcription factors of SREBF1, exerts a pivotal role in insulin resistance and insulin signaling pathways. SREBP 1c is competent to bind directly to and inhibit the activity of insulin receptor substrate 2 (IRS-2) (Shimano et al., 2007), which in turn participates in the IRS-2/PI3K/Akt pancreatic islet signaling pathway (Ide et al., 2004; Tsunekawa et al., 2011). A previous study has discovered that overexpression of SREBP-1c may induce islet mass deficiency and impaired insulin secretion (Kato et al., 2008). However, recent experimental research has indicated that SREBP1c regulates β-cell compensatory capacity in response to metabolic stress (Lee et al., 2019). SREBP1c knockout mice exhibited glucose intolerance and low insulin levels, and their β-cells had a reduced ability to proliferate and secrete insulin. In contrast, transplantation of islets overexpressing SREBP1c restored insulin levels and alleviated hyperglycemia. Reconceptualizing the regulatory mechanism of SREBF1 for β-cells could be a promising area of future research. ANPEP identified a remarkable allelic expression imbalance in islet tissues of type 2 diabetes, providing compelling support for type 2 diabetes susceptibility (Locke et al., 2015). ANPEP is involved in β-cell glutathione metabolism, and its expression is upregulated in diabetic patients. The triggering of unfolded protein response by dysregulation of glutathione metabolism is a potential mechanism of β-cell apoptosis and T2DM(Klyosova et al., 2023). BECN1 regulates the cellular autophagy process. In a BECN1 knockout mouse model, hyperactivation of autophagy degrades insulin granule vesicles in β-cells to reduce insulin secretion while suppressing endoplasmic reticulum stimulation in insulin-responsive cells and increasing insulin sensitivity (Yamamoto et al., 2018; Kuramoto and He, 2021). HNF1A haploinsufficiency is intimately correlated with the pathogenesis of maturity-onset diabetes of the young (MODY) and hypomorphic HNF1A variants increase the risk of type 2 diabetes mellitus (Qian et al., 2023). HNF1A regulates an extensive, highly histospecific genetic program in pancreatic islets and liver (Servitja et al., 2009), and deletion of HNF1A causes aberrant secretion of alpha and beta cells (Hermann et al., 2023; Qian et al., 2023). The N-glycosylation site of ST6GAL1 has a profound implication on diabetes susceptibility (Rudman et al., 2023). Variant loci of ST6GAL1 impact the risk of T2DM in cross-population research, and a population-based study in South Asia shows that genetic variation in ST6GAL1 is associated with pancreatic β-cell function (Kooner et al., 2011; Sabiha et al., 2021). PGF is a member of the vascular endothelial growth factor (VEGF) family. Some studies indicate that Serum levels of PGF are an excellent prognosticator of pre-eclampsia in women with gestational diabetes mellitus, and PGF levels are decreased in patients with gestational diabetes mellitus (Tsiakkas et al., 2015; Zen et al., 2020). It has been established that PGF modulates neovascularization and microvascular abnormalities in diabetic retinopathy (Zhao Y. et al., 2023). Further studies are awaited to confirm the correlation between PGF and diabetes. RXRA is a subtype of the Vitamin A-like X receptor (RXR), and RXR often binds to 9-cis retinoic acid (ATRA) to form a dimer and exert physiological functions. In response to ATRA stimulation, RXR upregulates the expression of SREBP1c, which collectively affects insulin secretion (Yang H.-Y. et al., 2022). USP7 encodes deubiquitinating enzymes and maintains the stability of pancreatic development. USP7 serves as a binding chaperone for phosphate inorganic transport protein 1 (PiT1), and deletion of PiT1 enables USP7 to bind persistently to IRS-1, preventing ubiquitination and promoting insulin pathway-regulated signaling in response to insulin stimulation (Forand et al., 2016). Furthermore, overexpression of USP7 in hepatic cells lowers blood glucose levels (Lee et al., 2013). In previous investigations, these therapeutic targets of baicalin have demonstrated potential for intervening in T2DM, yet the detailed molecular mechanisms still deserve further in-depth studies.

The regulatory pathways identified through enrichment analysis included the PI3K/AKT signaling pathway, autophagy, and apoptosis. Activation of the PI3K/Akt pathway can stimulate insulin secretion from pancreatic β-cells, whereas inhibition of Akt contributes to impaired insulin secretion (Bernal-Mizrachi et al., 2004). In liver and adipose tissue, PI3K/Akt is identically involved in mediating glucose homeostasis (Sajan et al., 2018). The notion that β-cell apoptosis elicits T2DM is supported by mounting evidence for apoptosis, a normal cellular process stabilizing alterations in β-cells clusters during pancreatic development (Finegood et al., 1995).

The decline in the quantity or dysfunction of pancreatic islet β-cells is the centerpiece of the mechanism that induces the dysregulation of glucose homeostasis that is responsible for the pathogenesis of diabetes mellitus. The islet microenvironment, which is collectively constituted by islet-cell interaction, directly or indirectly affects pancreatic islet β-cell function. The cellchat findings indicated that the intercellular cooperation of beta cells with Endothelial, Exocrine, and Delta cells performs a crucial role in pancreatic islet signaling pathways. β-cell outgrowth and development are influenced by pancreatic pericytes (PC). PCs sustain the structural integrity and functional normalization of the vasculature within the pancreatic islets, along with endothelial cells, constitute the microenvironment of the islets, and its depletion further drives a decrease in the expression of beta cell-associated developmental transcription factors, which impede β-cell maturation and differentiation (Sasson et al., 2016; Ahmed et al., 2024). Furthermore, Delta cells can reduce the glucose threshold of β-cells through a paracrine mechanism (Huang et al., 2024). In addition to endocrine cells, ductal, vesicular, and endothelial cells in exocrine tissues are closely intertwined with islet β-cell function, with a number of findings that damage to the exocrine pancreas is frequently comorbid with endocrine metabolic disorders (Zhi et al., 2019). Adenoalveolar cells may be essential to β-cell regeneration, and ductal cells possess the capability to differentiate into follicular cells (Li et al., 2014; Zhao H. et al., 2023). When pancreatic β-cells are compromised, exocrine cells might possess a tendency to polarize towards endocrine cells. An analysis using single-cell RNA sequencing on human pancreatic islet sections reveals that ductal and vesicular cells can be transformed into endocrine cells, providing new evidence for the possibility of β-cell regeneration studies (Doke et al., 2023). Based on exocrine-endocrine intercellular crosstalk, the discovery of drug targets in the role they play may offer new therapeutic approaches for the treatment of diabetes mellitus in the future.

Endothelial cells are probably the crucial component that affects the function of β-cells. Endocrine cells generate an angiogenic factor VEGFA in pancreatic development, and decreased or absent expression of VEGFA early in the process accounts for abnormalities in the pancreatic islet vascular system (Brissova et al., 2006). Its inactivation affects the proliferation of the adult β-cell population, which further contributes to the deficit of β-cell mass (Reinert et al., 2013). PGF is highly expressed in endothelial cells, with PGF facilitating angiogenesis in pathological states. Both PGF and VEGFA can activate tyrosine kinases, subsequently regulating the PI3K/AKT signaling pathway (Autiero et al., 2003). Inflammation-mediated endothelial cell impairment is also now recognized as one of the pathogenic mechanisms of diabetes. Inflammatory mediators are recruited intravascularly and increase pancreatic vascular permeability, accelerating the islet inflammatory response and islet cell destruction (Troullinaki et al., 2020). Persistently elevated glucose levels in the body might injure endothelial cells, resulting in declining vasodilatation and hastening the progression of diabetic panangiopathy (Shi et al., 2023; Wu et al., 2023).

This study possesses certain advantages. Firstly, this study innovatively employed Mendelian randomization and single-cell RNA sequencing to identify and analyze the targets of natural products according to our knowledge. This provides an analytical framework for the development of natural medicines and substantially shortens the drug development cycle. Secondly, the study utilized the largest diabetes GWAS and the most comprehensive statistical data on eQTLs to date, which enhanced the statistical efficacy and further guaranteed the applicability of the findings. Meanwhile, this study confirms the robustness of the results based on the mutual validation of multiple analyses. Enrichment and network analysis elucidated the functional properties and regulatory interrelationships of the therapeutic targets, while the potent binding activity from molecular docking assured the basis of action of the drugs and targets. Single-cell RNA sequencing data assessed the expression of these genes in the pancreas. Cellchat and pseudotemporal trajectories further probed cell-to-cell crosstalk and differentiation, augmenting the understanding of the pathogenesis of diabetes. Meanwhile, there are limitations to this study. First, the GWAS data were originated from European populations. This may limit the applicability of our findings to other ethnicities. MR effect estimates are susceptible to potential biases introduced by genetic background and population variation, so the generalization of the findings requires further research and validation. Second, MR does not completely generalize to real-world clinical trials, which simulate lifelong low-dose exposure to a drug and assume a linear relationship between exposure and outcome, whereas clinical trials typically study comparatively high doses of a drug over a much shorter period. Third, drugs also exhibit a broad spectrum of effects on their targets, and numerous off-target effects cannot be explored with MR. Finally, enrichment analyses are grounded in biological mechanisms clearly defined by previous research, yet unknown biological roles may not be accommodated. Molecular docking can theoretically boost the efficiency of virtual screening of drug targets to a great extent, but the specific effects in the clinic are yet to be verified.

## 5 Conclusion

In conclusion, our study identifies eight therapeutic targets of baicalin for diabetes from a genetic perspective and provides an analytical framework for natural product development. Expression of ANPEP, BECN1, HNF1A, and ST6GAL1 increased the risk of T2DM, whereas the decreased risk of T2DM was accompanied by expression of PGF, RXRA, SREBF1, and USP7. These findings contribute new insights and rationale for the clinical management and early intervention of diabetes, as well as the directions for future drug development in diabetes. More basic experimental and clinical research is warranted to delve into the role of these therapeutic targets and their molecular mechanisms.

## Data Availability

The raw datasets generated and analyzed in this study are available in the following repositories: eQTLs were obtained from the eQTLGen Consortium (https://eqtlgen.org/). Summary-level GWASs were derived from the DIAGRAM portal (https://diagram-consortium.org/) and the FinnGen R9 repository (https://r9.finngen.fi/), and single-cell RNA sequencing dataset GSE153855 originated from the Gene Expression Omnibus (GEO) database.
