# Supplementary material for: Integrating Mendelian randomization and single-cell RNA sequencing to identify therapeutic targets of baicalin for type 2 diabetes mellitus
Source: Front Pharmacol. 2024 Jul 26;15:1403943. doi: 10.3389/fphar.2024.1403943 (PMC11310057; doi:10.3389/fphar.2024.1403943)
Supplement: Supplementary file 1 [file DataSheet1.PDF]

STROBE-MR checklist of recommended items to address in reports of Mendelian randomization studies[1 2]

Item No.	Section	Checklist item	Page No.	Relevant text from manuscript
1	**TITLE and ABSTRACT**	Indicate Mendelian randomization (MR) as the study's design in the title and/or the abstract if that is a main purpose of the study	1 / 31	Integrating Mendelian randomization and single-cell RNA sequencing to identify therapeutic targets of baicalin for type 2 diabetes mellitus
	**INTRODUCTION**			
2	**Background**	Explain the scientific background and rationale for the reported study. What is the exposure? Is a potential causal relationship between exposure and outcome plausible? Justify why MR is a helpful method to address the study question	3 / 31	Expression quantitative trait locis (eQTLs) are genetic variations that regulate the expression of specific genes and offer clues for drug discovery(Chauquet et al., 2021). Since most risk variants for complex diseases exert their biological effect by influencing gene expression, integrating GWAS with eQTLs can aid in identifying potential drug targets. Incorporating human genetics and genomics may be one of the most efficient strategies to advance medication research, as therapies supported by genetic evidence are more likely to succeed in clinical trials and gain regulatory approval(Trajanoska et al., 2023). Currently, there is a lack of research investigating complementary and alternative treatments from a genome-wide perspective, and the identification and mechanistic exploration of natural drug targets may offer opportunities for preventing and treating T2DM.
3	**Objectives**	State specific objectives clearly, including pre-specified causal hypotheses (if any). State that MR is a method that, under specific assumptions, intends to estimate causal effects	3 / 31	Mendelian randomization (MR) is an epidemiological method that utilizes genetic variants as instrumental variables to assess causal relationships between exposures and outcomes. Its design is based on the random assortment of alleles during gamete formation, which is analogous to a natural randomized controlled experiment. Compared to traditional observational studies, this approach is more effective in minimizing bias due to confounding or reverse causation(Bowden and Holmes, 2019). This sophisticated design can more accurately capture the association between gene expression and complex disease phenotypes, providing a

				powerful tool for drug target discovery and validation(Võsa et al., 2021).<br><br>Applying large-scale GWASs and high-throughput single-cell RNA sequencing data, this study systematically integrates various bioinformatics techniques to identify the targets of baicalin in the treatment of type 2 diabetes and elucidate its mechanisms. It innovatively integrates natural products with modern genetics, providing novel insights for drug development and precision medicine.
	**METHODS**			
4	**Study design and data sources**	Present key elements of the study design early in the article. Consider including a table listing sources of data for all phases of the study. For each data source contributing to the analysis, describe the following:	4 / 31	The overall structure of this study based on guidelines for Mendelian randomization research was illustrated in Figure 1(Burgess et al., 2019; Skrivankova et al., 2021). Potential targets of baicalein were collected via multiple sources regarding natural products. We utilized the expression quantitative trait loci (eQTL) related to the drug targets in the eQTLGen Consortium as the exposure, and performed MR analysis with two independent T2D cohorts as the outcomes.
	a)	Setting: Describe the study design and the underlying population, if possible. Describe the setting, locations, and relevant dates, including periods of recruitment, exposure, follow-up, and data collection, when available.	5-6 / 31	The eQTL data in this study originated from the meta-analysis of the eQTLGen Consortium, and a detailed account of the data preparation could be found in the original publication(Võsa et al., 2021). The eQTLGen Consortium conducted an analysis of cis- and trans- expression quantitative trait loci using blood samples from 31,684 healthy European individuals across 37 independent cohorts, and a total of 19,250 genes were involved in the study. Cis-eQTL refered to single-nucleotide polymorphisms (SNPs) within 1Mb of the gene center and detected in at least two cohorts. The complete original data for cis-eQTL and allele frequency information can be downloaded from the eQTLGen portal (https://eqtlgen.org/).<br><br>The genome-wide association studies (GWASs) for type 2 diabetes were from the DIAGRAM consortium and the FinnGen R9 repository. Detailed information regarding subject recruitment and quality control could be found in the original publication(Mahajan et al., 2018; Kurki et al., 2023).

b)	Participants: Give the eligibility criteria, and the sources and methods of selection of participants. Report the sample size, and whether any power or sample size calculations were carried out prior to the main analysis	5-6 / 31	In the discovery phase, we selected the largest GWAS meta-analysis of European ancestry, involving 74,124 cases and 824,006 controls, available via DIAGRAM portal (https://diagram-consortium.org/)(Mahajan et al., 2018). The diagnosis of T2D was based on the clinical criteria of the American Diabetes Association or the World Health Organization, supplemented by healthcare registries, usage of antidiabetic medications, and valid self-reporting. Levels of GAD antibodies and fasting C-peptide, early insulin intervention, and family history were used to exclude patients with probable type 1 diabetes. The residual inflation of the summary statistics was corrected for through genomic control, and meta-analysis was adjusted for BMI. In the replication analysis, we employed a publicly available summary-level GWAS from the FinnGen R9 repository, including 38,657 cases and 310,131 controls(Kurki et al., 2023). T2D was defined according to the World Health Organization guidelines, with the inclusion and exclusion criteria under the International Classification of Diseases (ICD, https://r9.risteys.finngen.fi/) codes, specifically the 10th or 9th revision.
c)	Describe measurement, quality control and selection of genetic variants	6 / 31	We implemented a series of rigorous quality controls for cis-eQTLs to obtain reliable genetic instruments. Firstly, we identified common variants (minor allele frequency > 0.01) within a 100kb region surrounding the potential targets of baicalin with a significant threshold of P < 5×10e-8, ensuring that the instrumental variables could serve as proxies for exposure(Chen et al., 2022; Li et al., 2023). Secondly, utilizing a reference panel from the European population of the 1000 Genomes Project(1000 Genomes Project Consortium et al., 2015), we applied a linkage disequilibrium-based clustering with an r2=0.1 threshold within a 10000kb range to eliminate potential confounding effects generated by linkage between SNPs(Chen et al., 2022). Thirdly, we computed the F-statistic for each instrumental variable to estimate their strength (R2 = 2×EAF×(1-EAF) × beta2; F = R2 × (N-2)/(1-R2)), and excluded SNPs with an F-statistic below 10 to eliminate bias from weak instrumental

					variables(Burgess et al., 2011; Papadimitriou et al., 2020). Fourthly, Steiger filtering was applied to remove drug targets where SNPs accounted for a larger fraction of the variation in T2D risk than gene expression to ensure unidirectionality of causality. In addition, we removed palindromic SNPs with uncertain strands and SNPs with non-concordant alleles to avoid any potential errors in allele determination and provide accurate causality assessments.
		d)	For each exposure, outcome, and other relevant variables, describe methods of assessment and diagnostic criteria for diseases	5-6 / 31	The eQTLGen Consortium conducted an analysis of cis- and trans- expression quantitative trait loci using blood samples from 31,684 healthy European individuals across 37 independent cohorts, and a total of 19,250 genes were involved in the study. Cis-eQTL refered to single-nucleotide polymorphisms (SNPs) within 1Mb of the gene center and detected in at least two cohorts.<br><br>The diagnosis of T2D was based on the clinical criteria of the American Diabetes Association or the World Health Organization, supplemented by healthcare registries, usage of antidiabetic medications, and valid self-reporting. Levels of GAD antibodies and fasting C-peptide, early insulin intervention, and family history were used to exclude patients with probable type 1 diabetes.<br><br>T2D was defined according to the World Health Organization guidelines, with the inclusion and exclusion criteria under the International Classification of Diseases (ICD, https://r9.risteys.finngen.fi/) codes, specifically the 10th or 9th revision.
		e)	Provide details of ethics committee approval and participant informed consent, if relevant	5 / 31	Data was derived from aggregated meta-GWASs and publicly available eQTL statistics, with original studies authorized by their respective institutional review boards and ethics committees, and all participants granted informed consent.
5	**Assumptions**		Explicitly state the three core IV assumptions for the main analysis (relevance, independence and exclusion restriction) as well assumptions for any additional or sensitivity analysis	6 / 31	Mendelian randomization study must fulfil three key assumptions: (1) Instrumental variables (IVs) derived from genetic variation should be tightly associated with the exposure; (2) confounding factors are independent to the selected IVs; (3) The instrumental variables solely impact the outcome through the exposure.

6	**Statistical methods: main analysis**		Describe statistical methods and statistics used	6 / 31	In the primary analysis, if only one eQTL for the drug target was available, we employed the Wald method that calculated the coefficient ratio for the outcomes and exposures. When two or more instrumental variables were available, the meta-analysis integrated with the Wald ratio on each SNP was performed using inverse-variance weighted (IVW), MR-Egger, weighted median and maximum likelihood methods. The primary assessment was completed applying the IVW approach, yielding general estimates through meta-analysis in combination with Wald ratios for each SNP(Burgess et al., 2017). Compared to the fixed effects model, The IVW method with multiplicative random effects model (REM) could guarantee statistical efficacy even in the presence of weaker random effects(Burgess et al., 2019). In comparison to the IVW approach, the other methods exhibited relatively inferior statistical efficacy. Consequently, they were solely applied to corroborate the general direction of the primary method.
		a)	Describe how quantitative variables were handled in the analyses (i.e., scale, units, model)	5 / 31	The residual inflation of the summary statistics was corrected for through genomic control, and meta-analysis was adjusted for BMI.
		b)	Describe how genetic variants were handled in the analyses and, if applicable, how their weights were selected	6 / 31	In the primary analysis, if only one eQTL for the drug target was available, we employed the Wald method that calculated the coefficient ratio for the outcomes and exposures. When two or more instrumental variables were available, the meta-analysis integrated with the Wald ratio on each SNP was performed using inverse-variance weighted (IVW), MR-Egger, weighted median and maximum likelihood methods. The primary assessment was completed applying the IVW approach, yielding general estimates through meta-analysis in combination with Wald ratios for each SNP(Burgess et al., 2017). Compared to the fixed effects model, The IVW method with multiplicative random effects model (REM) could guarantee statistical efficacy even in the presence of weaker random effects(Burgess et al., 2019). In comparison to the IVW approach, the other methods exhibited relatively inferior statistical efficacy. Consequently, they were solely applied

					to corroborate the general direction of the primary method.
		c)	Describe the MR estimator (e.g. two-stage least squares, Wald ratio) and related statistics. Detail the included covariates and, in case of two-sample MR, whether the same covariate set was used for adjustment in the two samples	6 / 31	In the primary analysis, if only one eQTL for the drug target was available, we employed the Wald method that calculated the coefficient ratio for the outcomes and exposures. When two or more instrumental variables were available, the meta-analysis integrated with the Wald ratio on each SNP was performed using inverse-variance weighted (IVW), MR-Egger, weighted median and maximum likelihood methods.
		d)	Explain how missing data were addressed	Not applicable	
		e)	If applicable, indicate how multiple testing was addressed	7 / 31	Bonferroni correction was implemented in the discovery cohort to define the significance threshold for multiple testing. Drug targets with a P-value < 1.00e-4 (0.05/499) were defined as significant. Sensitivity analysis was conducted on the initially identified potential targets, and validation was performed in a replication cohort. The significance threshold for the validation phase was established at 0.0016 (0.05/31)
7	**Assessment of assumptions**		Describe any methods or prior knowledge used to assess the assumptions or justify their validity	7 / 31	To further ascertain the potential shared genetic effects between drug targets and T2D risk, we conduct colocalization analysis using the R package coloc (version 5.2.3). Colocalization analysis requires the inclusion of all SNPs within a genomic region, providing a comprehensive method for utilizing genetic information to evaluate the therapeutic targets of baicalin(Wallace, 2020). We applied a prior probability of 1e-04 for baicalein targets (H1) and T2D phenotypes (H2), while setting the prior probability to 1e-05 for an individual variant being associated with both gene expression and T2D risk (H4). For each potential target of baicalin, we included SNPs within a range of ±1Mb from the gene start and end points. The significance criterion for colocalization was defined as PP.H4 greater than 0.80.
8	**Sensitivity analyses and additional analyses**		Describe any sensitivity analyses or additional analyses performed (e.g. comparison of effect estimates from different approaches, independent replication, bias analytic techniques, validation of instruments, simulations)	7 / 31	We applied the IVW method and Egger regression to detect heterogeneity for baicalin targets containing two or more instrumental variables. Heterogeneity was quantified using Cochran's Q

					test, with $p < 0.05$ indicating apparent heterogeneity among instrumental variables. The MR-Egger intercept was employed to evaluate the existence of pleiotropy across instrumental variables, and no meaningful horizontal pleiotropy was observed if $P > 0.05$.
9	**Software and pre-registration**				
		a)	Name statistical software and package(s), including version and settings used	6 / 31	We utilized the "TwoSampleMR" package (version 0.5.7) in R software (version 4.2.1) for the MR procedure and sensitivity analysis(Hemani et al., 2018).
		b)	State whether the study protocol and details were pre-registered (as well as when and where)	Not applicable	
	**RESULTS**				
10	**Descriptive data**				
		a)	Report the numbers of individuals at each stage of included studies and reasons for exclusion. Consider use of a flow diagram	9 / 31	We acquired potential targets of baicalin from multiple sources to ensure the comprehensive scope of the study. In particular, there were 58 baicalin-related targets in BATMAN-TCM 2.0 database, 86 targets in SymMap V2, 66 targets in TCMIP v2.0, 258 targets in ChEMBL, 53 targets in CTD, 28 targets in STITCH, 297 targets in PharmMapper, 100 targets in SwissTarget Prediction, 94 targets in SuperPred, and 36 targets in SEA. After merging, deduplication and standardization, there are a total of 808 potential drug targets for baicalin. We obtained cis-eQTLs tightly associated with baicalin targets in a reliable ($P < 5e\times10-8$) and independent ($r2< 0.1$, kb = 10,000) manner from the eQTLGen consortium. After a series of quality control measures including exclusion based on F-statistics and Steiger's filtering, we selected the eQTL from the final set of 499 target genes.
		b)	Report summary statistics for phenotypic exposure(s), outcome(s), and other relevant variables (e.g. means, SDs, proportions)	Not applicable	
		c)	If the data sources include meta-analyses of previous studies, provide the assessments of heterogeneity across these studies	Not applicable	

		d)	For two-sample MR:<br>i. Provide justification of the similarity of the genetic variant-exposure associations between the exposure and outcome samples<br>ii. Provide information on the number of individuals who overlap between the exposure and outcome studies	4-6 / 31	Subjects included in the discovery and validation cohorts were restricted to European descent to minimize potential bias in population stratification.<br>The probability of overlap in population selection between the exposure and outcome was minimal.
11	**Main results**				
		a)	Report the associations between genetic variant and exposure, and between genetic variant and outcome, preferably on an interpretable scale	9-11 / 31	During the discovery phase, we selected the largest T2D GWAS currently available as the outcome and conducted MR analysis using eQTLs for potential targets of baicalin. Applying the Wald ratio or IVW method with a multiplicative random effects model, a total of 35 baicalin targets remained significantly ($P < 1.00e-4$) associated with T2D risk after Bonferroni correction (Figure 2A). DHODH, HSD17B1, and ODC1 were excluded from the MR-Egger method due to inconsistent causal estimates in the MR-Egger compared with other methods (Supplementary Table 1). CFD was excluded due to unaccountable heterogeneity. Despite the presence of heterogeneity in ANPEP, BECN1, P2RX4, and ST6GAL1, their causal estimates remained significant after multiple tests in the weighted mean method ($P < 1.00e-4$), indicating relatively robust results (Supplementary Table 2). The Egger intercept test demonstrated no apparent horizontal pleiotropy. After the above sensitivity analysis, 31 genes were included for subsequent validation (Figure 2B). Genetic prediction indicated elevated levels of AKT2, AMD1, ANPEP, BECN1, CA4, CLC, F10, FGF2, HNF1A, MPO, MYC, NOS3, P2RX4, ST6GAL1 and USP7 were associated with increased risk of T2D, while the concentrations of CASP1, CD38, CDA, DHFRL1, FKBP1B, FPGS, HES1, KDM5A, NCOA1, NFKB1, PGF, PRMT3, RELA, RXRA, SREBF1, and UCK2 exhibited a negative correlation with T2D risk. These associations are consistent across other approaches, and the results of genome-wide MR in the discovery phase are presented in Supplementary Table 1.

		b)	Report MR estimates of the relationship between exposure and outcome, and the measures of uncertainty from the MR analysis, on an interpretable scale, such as odds ratio or relative risk per SD difference	9-11 / 31	Employing GWAS from the FinnGen R9 repository for validation, we conducted a replication analysis of the potential targets of baicalin and performed MR analysis in a manner consistent with the discovery cohort. We evaluated the potential causal relationship between potential targets of baicalein and the risk of T2DM by applying the Wald ratio or the IVW method with a multiplicative random effects model (Supplementary Table 3), and 8 baicalin-related targets remained significant ($P < 0.0016$) after Bonferroni correction (Figure 3). Specifically, the elevated expression of ANPEP, BECN1, HNF1A, and ST6GAL1 was associated with an increased risk of T2D, while the expression of PGF, RXRA, SREBF1, and USP7 decreased the risk of T2D. These targets exhibited identical causal effects across the four MR methods, and their impact on the outcome in the validation cohort remained consistent with that observed in the discovery cohort. ST6GAL1 demonstrated heterogeneity among SNPs in the Cochran's Q test, yet it exhibited statistical significance in the weighted mean method (Supplementary Table 4). The Egger intercept test revealed no significant pleiotropy for any target.
		c)	If relevant, consider translating estimates of relative risk into absolute risk for a meaningful time period	Not applicable	
		d)	Consider plots to visualize results (e.g. forest plot, scatterplot of associations between genetic variants and outcome versus between genetic variants and exposure)	9-11 / 31	Figure 2 and 3
12	**Assessment of assumptions**				
		a)	Report the assessment of the validity of the assumptions	9-11 / 31	After a series of quality control measures including exclusion based on F-statistics and Steiger's filtering, we selected the eQTL from the final set of 499 target genes.<br>DHODH, HSD17B1, and ODC1 were excluded from the MR-Egger method due to inconsistent causal estimates in the MR-Egger compared with other methods (Supplementary Table 1). CFD was excluded due to unaccountable heterogeneity. Despite the presence of heterogeneity in ANPEP, BECN1, P2RX4, and ST6GAL1, their causal

					estimates remained significant after multiple tests in the weighted mean method (P < 1.00e-4), indicating relatively robust results (Supplementary Table 2). The Egger intercept test demonstrated no apparent horizontal pleiotropy.<br>ST6GAL1 demonstrated heterogeneity among SNPs in the Cochran's Q test, yet it exhibited statistical significance in the weighted mean method (Supplementary Table 4). The Egger intercept test revealed no significant pleiotropy for any target.
		b)	Report any additional statistics (e.g., assessments of heterogeneity across genetic variants, such as I^2, Q statistic or E-value)	9-11 / 31	Supplementary Table 2 and 4
13	**Sensitivity analyses and additional analyses**				
		a)	Report any sensitivity analyses to assess the robustness of the main results to violations of the assumptions	9-11 / 31	DHODH, HSD17B1, and ODC1 were excluded from the MR-Egger method due to inconsistent causal estimates in the MR-Egger compared with other methods (Supplementary Table 1). CFD was excluded due to unaccountable heterogeneity. Despite the presence of heterogeneity in ANPEP, BECN1, P2RX4, and ST6GAL1, their causal estimates remained significant after multiple tests in the weighted mean method (P < 1.00e-4), indicating relatively robust results (Supplementary Table 2). The Egger intercept test demonstrated no apparent horizontal pleiotropy.<br>ST6GAL1 demonstrated heterogeneity among SNPs in the Cochran's Q test, yet it exhibited statistical significance in the weighted mean method (Supplementary Table 4). The Egger intercept test revealed no significant pleiotropy for any target.
		b)	Report results from other sensitivity analyses or additional analyses	9-11 / 31	DHODH, HSD17B1, and ODC1 were excluded from the MR-Egger method due to inconsistent causal estimates in the MR-Egger compared with other methods (Supplementary Table 1). CFD was excluded due to unaccountable heterogeneity. Despite the presence of heterogeneity in ANPEP, BECN1, P2RX4, and ST6GAL1, their causal estimates remained significant after multiple tests

				in the weighted mean method (P < 1.00e-4), indicating relatively robust results (Supplementary Table 2). The Egger intercept test demonstrated no apparent horizontal pleiotropy.<br><br>ST6GAL1 demonstrated heterogeneity among SNPs in the Cochran's Q test, yet it exhibited statistical significance in the weighted mean method (Supplementary Table 4). The Egger intercept test revealed no significant pleiotropy for any target.
		c) Report any assessment of direction of causal relationship (e.g., bidirectional MR)	6 / 31	Fourthly, Steiger filtering was applied to remove drug targets where SNPs accounted for a larger fraction of the variation in T2D risk than gene expression to ensure unidirectionality of causality.
		d) When relevant, report and compare with estimates from non-MR analyses	16 / 31	The heatmap demonstrated the expression of baicalin-related targets in different cell types (Fig. 7B) and in each cell (Fig. 7C), and the eight targets have a wide range of expression in the pancreatic cells. In particular, SREBF1and ST6GAL1 were highly expressed in beta cells, so were HNF1A and SREBF1 in gamma cells, ANPEP in exocrine cells, RXRA in macrophage cells, ST6GAL1, BECN1 and PGF in endothelial cells, and USP7 in mast cells.
		e) Consider additional plots to visualize results (e.g., leave-one-out analyses)	Not applicable	
	**DISCUSSION**			
14	**Key results**	Summarize key results with reference to study objectives	20 / 31	Applying eQTLs for baicalin-related targets, we performed MR analysis in discovery and replication cohorts and identified eight therapeutic targets causally associated with T2DM: ANPEP, BECN1, HNF1A, ST6GAL1, PGF, RXRA, SREBF1, USP7.
15	**Limitations**	Discuss limitations of the study, taking into account the validity of the IV assumptions, other sources of potential bias, and imprecision. Discuss both direction and magnitude of any potential bias and any efforts to address them	23 / 31	Meanwhile, there are limitations to this study. First, the GWAS data were originated from European populations. This may limit the applicability of our findings to other ethnicities. MR effect estimates are susceptible to potential biases introduced by genetic background and population variation, so the generalization of the findings requires further research and validation. Second, MR does not completely generalize to real-world

				clinical trials, which simulate lifelong low-dose exposure to a drug and assume a linear relationship between exposure and outcome, whereas clinical trials typically study comparatively high doses of a drug over a much shorter period. Third, drugs also exhibit a broad spectrum of effects on their targets, and numerous off-target effects cannot be explored with MR. Finally, enrichment analyses are grounded in biological mechanisms clearly defined by previous research, yet unknown biological roles may not be accommodated. Molecular docking can theoretically boost the efficiency of virtual screening of drug targets to a great extent, but the specific effects in the clinic are yet to be verified.
16	**Interpretation**			
		a)	Meaning: Give a cautious overall interpretation of results in the context of their limitations and in comparison with other studies	20-21 / 31	SREBF1, known as SREBP1, engages in the encoding of sterol regulatory element binding proteins. With strong colocalization support, SREBF1 was highly expressed in β-cells and exhibited important interacting properties in the PPI network. SREBP1c, one of the transcription factors of SREBF1, exerts a pivotal role in insulin resistance and insulin signaling pathways. SREBP 1c is competent to bind directly to and inhibit the activity of insulin receptor substrate 2 (IRS-2)(Shimano et al., 2007) , which in turn participates in the IRS-2/PI3K/Akt pancreatic islet signaling pathway(Ide et al., 2004; Tsunekawa et al., 2011). A previous study has discovered that overexpression of SREBP-1c may induce islet mass deficiency and impaired insulin secretion(Kato et al., 2008). However, recent experimental research has indicated that SREBP1c regulates β-cell compensatory capacity in response to metabolic stress(Lee et al., 2019). SREBP1c knockout mice exhibited glucose intolerance and low insulin levels, and their β-cells had a reduced ability to proliferate and secrete insulin. In contrast, transplantation of islets overexpressing SREBP1c restored insulin levels and alleviated hyperglycemia. Reconceptualizing the regulatory mechanism of SREBF1 for β-cells could be a promising area of future research. ANPEP identified a remarkable allelic expression imbalance in islet tissues of type 2 diabetes,

providing compelling support for type 2 diabetes susceptibility(Locke et al., 2015). ANPEP is involved in β-cell glutathione metabolism, and its expression is upregulated in diabetic patients. The triggering of unfolded protein response by dysregulation of glutathione metabolism is a potential mechanism of β-cell apoptosis and T2DM(Klyosova et al., 2023). BECN1 regulates the cellular autophagy process. In a BECN1 knockout mouse model, hyperactivation of autophagy degrades insulin granule vesicles in β-cells to reduce insulin secretion while suppressing endoplasmic reticulum stimulation in insulin-responsive cells and increasing insulin sensitivity(Yamamoto et al., 2018; Kuramoto and He, 2021). HNF1A haploinsufficiency is intimately correlated with the pathogenesis of maturity-onset diabetes of the young (MODY) and hypomorphic HNF1A variants increase the risk of type 2 diabetes mellitus(Qian et al., 2023). HNF1A regulates an extensive, highly histospecific genetic program in pancreatic islets and liver(Servitja et al., 2009), and deletion of HNF1A causes aberrant secretion of alpha and beta cells(Hermann et al., 2023; Qian et al., 2023). The N-glycosylation site of ST6GAL1 has a profound implication on diabetes susceptibility(Rudman et al., 2023). Variant loci of ST6GAL1 impact the risk of T2DM in cross-population research, and a population-based study in South Asia shows that genetic variation in ST6GAL1 is associated with pancreatic β-cell function(Kooner et al., 2011; Sabiha et al., 2021). PGF is a member of the vascular endothelial growth factor (VEGF) family, and suppressing PGF reduces neovascularization and microvascular abnormalities in diabetic retinopathy(Nguyen et al., 2018; Zhao et al., 2023b). Serum levels of PGF are an excellent prognosticator of pre-eclampsia in women with gestational diabetes mellitus(Zen et al., 2020). RXRA is a subtype of the Vitamin A-like X receptor (RXR), and RXR often binds to 9-cis retinoic acid (ATRA) to form a dimer and exert physiological functions. In response to ATRA stimulation, RXR upregulates the expression of SREBP1c, which collectively affects insulin secretion(Yang et al., 2022a). USP7 encodes deubiquitinating enzymes and maintains the

					stability of pancreatic development. USP7 combines with IRS-1 upon insulin stimulation to block both ubiquitination and insulin pathway signaling(Forand et al., 2016). Nevertheless, overexpression of USP7 in hepatic cells lowers blood glucose levels(Lee et al., 2013). In previous investigations, these therapeutic targets of baicalin have demonstrated potential for intervening in T2DM, yet the detailed molecular mechanisms still deserve further in-depth studies.
		b)	Mechanism: Discuss underlying biological mechanisms that could drive a potential causal relationship between the investigated exposure and the outcome, and whether the gene-environment equivalence assumption is reasonable. Use causal language carefully, clarifying that IV estimates may provide causal effects only under certain assumptions	21 / 31	The regulatory pathways identified through enrichment analysis included PI3K/AKT signaling pathway, autophagy, and apoptosis. Activation of PI3K/Akt pathway can stimulate insulin secretion from pancreatic β-cells, whereas inhibition of Akt contributes to impaired insulin secretion(Bernal-Mizrachi et al., 2004). In liver and adipose tissue, PI3K/Akt is identically involved in mediating glucose homeostasis(Sajan et al., 2018). The notion that β-cell apoptosis elicits T2DM is supported by mounting evidence for apoptosis, a normal cellular process stabilizing alterations in β-cells clusters during pancreatic development(Finegood et al., 1995).
		c)	Clinical relevance: Discuss whether the results have clinical or public policy relevance, and to what extent they inform effect sizes of possible interventions	20-21 / 31	Available in the discussion section.
17	**Generalizability**		Discuss the generalizability of the study results (a) to other populations, (b) across other exposure periods/timings, and (c) across other levels of exposure	23 / 31	First, the GWAS data were originated from European populations. This may limit the applicability of our findings to other ethnicities. MR effect estimates are susceptible to potential biases introduced by genetic background and population variation, so the generalization of the findings requires further research and validation.
	**OTHER INFORMATION**				
18	**Funding**		Describe sources of funding and the role of funders in the present study and, if applicable, sources of funding for the databases and original study or studies on which the present study is based	24 / 31	This research was funded by the National Natural Science Foundation of China (grant number: 82274419) and the Natural Science Foundation of Guangdong Province (grant number: 2020A1515010775).
19	**Data and data sharing**		Provide the data used to perform all analyses or report where and how the data can be accessed, and reference these sources in the article. Provide the statistical	23 / 31	The raw datasets generated and analyzed in this study are available in the following repositories:

		code needed to reproduce the results in the article, or report whether the code is publicly accessible and if so, where		eQTLs data were obtained from eQTLGen Consortium (https://eqtlgen.org/). Summary-level GWASs were derived from the DIAGRAM portal (https://diagram-consortium.org/) and the FinnGen R9 repository (https://r9.finngen.fi/), and single-cell RNA sequencing dataset GSE153855 originated from the Gene Expression Omnibus (GEO) database.
20	**Conflicts of Interest**	All authors should declare all potential conflicts of interest	24 / 31	The authors declare that they have no conflicts of interest.

This checklist is copyrighted by the Equator Network under the Creative Commons Attribution 3.0 Unported (CC BY 3.0) license.

1. Skrivankova VW, Richmond RC, Woolf BAR, Yarmolinsky J, Davies NM, Swanson SA, et al. Strengthening the Reporting of Observational Studies in Epidemiology using Mendelian Randomization (STROBE-MR) Statement. JAMA. 2021;under review.

2. Skrivankova VW, Richmond RC, Woolf BAR, Davies NM, Swanson SA, VanderWeele TJ, et al. Strengthening the Reporting of Observational Studies in Epidemiology using Mendelian Randomisation (STROBE-MR): Explanation and Elaboration. BMJ. 2021;375:n2233.
